# Nutrients utilization and enteric methane emission in zebu cattle fed low quality forages

**DOI:** 10.1016/j.vas.2025.100511

**Published:** 2025-09-18

**Authors:** Gérard Xavier Gbenou, Luc Hippolyte Dossa, Denis Bastianelli, Ollo Sib, Laurent Bonnal, Cécile Martin, Mohamed Habibou Assouma

**Affiliations:** a Laboratoire des Sciences Animales, Faculté des Sciences Agronomiques, Université d’Abomey-Calavi 526 Cotonou, Benin; b SELMET, University of Montpellier, CIRAD, INRAE, Institut Agro, Montpellier, France; c CIRAD, UMR SELMET, dP ASAP, Bobo Dioulasso, Burkina Faso; d Centre International de Recherche Développement sur l’Elevage en zone Subhumide 454 Bobo-Dioulasso, Burkina Faso; e Université Clermont Auvergne, INRAE, VetAgro Sup, UMR 1213 Herbivores 63122 Saint-Genès-Champanelle, France

**Keywords:** Tropical forage, Feed supply, Greenhouse gas, Ruminant livestock, Emission factor, Sub-Saharan Africa

## Abstract

During the dry season in sub-Saharan Africa, ruminants are commonly fed low-quality forages, such as rangeland fodder and harvested grasses. This study evaluated the effects of four such forages - rangeland fodder (RF), *Brachiaria ruziziensis* (Bruzi), *Andropogon gayanus* (Agaya), and *Panicum maximum* (Pmaxi), all harvested at maturity (24 months) on feed intake, diet digestibility, and enteric methane (eCH₄) emissions. Ten Sudanese Fulani zebu steers (27.7 ± 4.6 months of age, 143.7 ± 15.7 kg of initial body weight - BW) were fed each forage at 2.3 % and 3.2 % of body weight (dry matter – DM basis) over three-week periods, two weeks of adaptation and one week of data collection. The forages differed significantly in chemical composition, affecting feed intake, diet digestibility, and eCH₄ emissions. The Pmaxi, with superior nutritional quality, was the most consumed and had the highest apparent digestibility (0.49; *P* < 0.001). At low-quality stage, all the forages had similar eCH_4_ yield potential (28.5 - 31.6 g/kg DM intake; *P* = 0.486). Methane conversion rate (Ym) ranged from 8.6 % in Pmaxi to 9.6 % of gross energy intake in Agaya; however, the variation was not statistically significant. Feeding ruminants over ad libitum low-quality forages did not affect their feed intake, diet apparent digestibility and eCH_4_ emissions. Measuring eCH_4_ emissions induced by these forages at different growth stages can help identify their optimal use for promoting both feed and environmental sustainability.

## Highlights

• In the dry season, ruminants in sub-Saharan Africa depend on nutritionally poor forages.
• Variation in feed quality affects intake, digestibility, and enteric methane emissions.
• Supplying feed ad libitum doesn’t improve intake or reduce emissions per kg of DMI.
• Improving forage quality is key to increasing intake and mitigating methane emissions.

## 1 Introduction

Livestock systems in the Sahel are predominantly pastoral and rely heavily on rangeland fodder (Diawara et al., 2017). Rangeland productivity is shaped by seasonal variation, grazing pressure, stocking density, cropland expansion, and natural factors (Kiema et al., 2014; Koutou et al., 2016). During the dry season, forage quality declines due to reduced nitrogen content (Koutou et al., 2016) and elevated levels of fiber, specifically neutral detergent fiber (NDF) and acid detergent fiber (ADF), resulting in an inadequate nutrient supply to meet the dietary requirements of ruminants (Obulbiga et al., 2015). Beyond rangelands, a wide range of forages and crop residues are used in Sub Saharan Africa (SSA) to feed livestock (Amole, Augustine, Balehegn, & Adesogoan, 2022; Okoli et al., 2003). They are typically harvested and stored at maturity, despite their low quality, to mitigate feed shortages later in the season. Several species, including *Panicum maximum, Andropogon gayanus*, and *Brachiaria ruziziensis,* are now commonly used in ruminant diets across SSA by both farmers and research institutions (Klein et al., 2014).

Forage diversity increases feed availability and animal production, supporting farmer profitability, but also leads to variability in enteric methane (eCH_4_) emissions. In contrast, feeding ruminants low-quality forages tends to reduce dry matter intake (DMI), primarily due to nitrogen limitations in the rumen and the consequent decline in microbial activity (Gbenou et al., 2024a). This raises the question of whether offering forage above basal levels could stimulate intake and improve diet intake quality through animal selection. However, data on the relationship between forage quality and eCH_4_ emissions under Sahelian and Sudano-Sahelian conditions remain limited, especially for local ruminant breeds consuming regionally representative forages, as opposed to temperate production systems (Kering et al., 2024; Meo-Filho et al., 2023). A better understanding of how low-quality forage diversity and feed supply levels affect eCH_4_ emissions is essential for developing regionally appropriate mitigation strategies. Therefore, this study investigates how the diversity of low-quality forages and feed supply above basal affect intake and eCH_4_ emissions in the Sahel. Specifically, it aims to evaluate the nutritional quality, intake and digestibility, and methanogenic potential of these forages hays in Sudanese Fulani zebu cattle.

## 2 Methodology

### 2.1 Study site

The study was carried out at the CIRDES experimental station in Bobo-Dioulasso in South-Western Burkina Faso (11° 10′37″N and 4°17′52″W) during the hot dry season (March to June 2021) characterized by an ambient temperature of 34.1 °C and rainfall of 169 mm. The protocol was approved by the CIRDES ethics committee (N°006/March/2021/CE-CIRDES).

### 2.2 Animals, feed and experimental design

The study involved ten Sudanese Fulani zebu steers (27.7 ± 4.6 months, 143.7 ± 15.7 kg initially) kept in a 9 m^2^ individual pen within a ventilated experimental barn. Pens had concrete floors without bedding and were cleaned every morning.

The tested forages included natural rangeland fodder (RF), *P. maximum* C1 (Pmaxi), *A. gayanus* (Agaya), and *B. ruziziensis* (Bruzi). The RF, dominated by *A. gayanus* Kunth, *Hyparrhenia hirta,* and *Pennisetum pedicellatum*, was harvested fresh every 2 days during the hot dry season from a pastoral area (rural commune of Bama) located 20 km from Bobo-Dioulasso (11° 23′ 59″ north, 4° 25′ 46″ west) and stored immediately. Cultivated grasses were grown at the Institut National de l’Environnement et des Recherches Agricoles (INERA, Burkina Faso), harvested at maturity (24 weeks), dried on site, and bundled.

Animals were fed each forage at two levels, 2.3 and 3.2 % of their BW on a DM basis, resulting in eight feeding trials (two per forage). Lick stones and water were provided ad libitum. Daily rations were offered in two meals served at 8:30 h and 16:30 h. To facilitate eCH_4_ measurement, a bait composed of RF and molasses (90:10 DM ratio) was distributed via the automatic feeder of a GreenFeed® system (GF – ID: 252, C-Lock Inc., Rapid City, South Dakota, USA). The RF was ground through a 1-mm mesh sieve, mixed with molasses, and pelleted (8 mm diameter).

### 2.3 Measurement and sample collection

Each trial lasted 3 weeks with 2 weeks of adaptation to the feed and 1 week of data (intake, excreted faeces, apparent digestibility and eCH_4_ emissions) collection.

#### 2.3.1 Body weight, intake and apparent digestibility

Initial BW, forage intake, and digestibility were assessed according to the methodology of Gbenou et al. (2024a, 2024b). The BW was recorded at the start and end of each trial. Voluntary feed intake was measured daily by weighing the feed offered and refused for each animal. Representative samples (300 g) of both offered diets and refusals were collected and kept individually, along with a sample of the GF bait. Total faecal output was collected daily using a faecal harness fitted to each animal and emptied twice a day. A daily representative fecal of 800 g was taken per animal. Per trial and forage, seven offered feed samples, 70 refusals samples, and 70 fecal samples were collected. All samples were immediately dried at 55 °C for 72 h and ground to 1 mm (SM 100, Retsch GmbH, Hann, Germany) for chemical analysis.

#### 2.3.2 Enteric methane emissions

Emissions of eCH_4_ were measured with the GF at different times of the day based on animal behavior, as described by Gbenou et al. (2024a, 2024b): 6:30 h (fasting), 10:00 h (just after eating), 14:00 h (during rumination) and 18:00 h (just after eating and at sunset). An additional measurement was taken at 00:00 h (during total rest) on day 7 of each forage trial. Each animal completed the 29 GF visits per trial, spending an average of 3min±07 s (Min = 2 min 01 s, Max = 5 min 24 s) per visit. The GF unit was set to deliver a drop of 34 ± 1.8 g of bait per minute. The bait, composed of RF and molasses to minimize its effect on diet composition. The GF unit was automatically calibrated daily at 4:00 h using a certified gas mixture (CH_4_: 509.4 ppm, CO_2_: 4993 ppm, H_2_: 10.10 ppm, and O_2_: 21.01 ppm; Liquid air, C-Lock, USA). CO_2_ recovery tests were performed twice weekly (at the beginning and end of each trial), and the filter was replaced as needed (e.g. when airflow dropped below 27 L/s). Across the study, average airflow was 38.6 ± 4.9 L/s (Min = 22.94, Max = 41.11), with a mean recovery rate of 96.5 ± 1.7 % (Min = 92.97, Max = 99.0), and wind direction averaging 136.2 ± 42.18 degrees (Min = 5.07, Max = 353.85).

#### 2.3.3 Determination of chemical composition of forages and faeces

The chemical composition of offered and refused forages and baits (ash, CP, NDF, ADF, ADL and GE) as well as that of faeces (ash, N, NDF, ADF, ADL) was determined by near-infrared spectroscopy (NIRS) as described by Gbenou et al. (2024a, 2024b). NIRS spectra were obtained on a reflectance spectrometer (Tango model, Bruker Optik GmbH, Ettlingen, Germany) which captures spectra between 11,536 and 3952 cm step. Prediction models, developed from 1890 forage and 690 faecal samples and validated by reference wet chemistry, were used for nutrient estimation. Ash content was determined by incineration at 550 °C for 5 h in a muffle furnace. Fiber fractions (NDF, ADF, ADL) were analysed using an Ankom fiber analyser (Ankom® Tech. Co., Fairport, NY, USA). NDF and ADF were corrected for ash in the ADL residue. Total nitrogen (N) was determined by the Kjeldahl method, and CP content calculated as N x 6.25. In vitro digestibility of dry matter (IVDMD) and organic matter (IVOMD) was determined using the pepsin-cellulase method (Aufrère et al., 2007). Gross energy (GE) of feed offered and refusals was determined by bomb calorimetry (IKA calorimeter model C2000; IKA-Werke GmbH, Staufen, Germany). Intake composition was derived from offered and refused fractions. Feed and diet compositions are presented in Table 1.

**Table 1:** Chemical composition and nutritional quality of forage hay offered to Sudanese Fulani zebu steers.

Item	RF	Bruzi	Pmaxi	Agaya	SEM	P-value
DM (g/kg CM)	942.8 ^A^	870.0 ^B^	930.7 ^A^	875.4 ^B^	0.00	<0.001
OM (g/kg DM)	870.1 ^B^	920.3 ^A^	884.6 ^B^	908.2 ^A^	0.37	<0.001
CP (g/kg DM)	27.5 ^B^	24.1 ^C^	37.2 ^A^	20.7 ^D^	0.09	<0.001
NDF (g/kg DM)	735.4 ^C^	773.1 ^B^	725.7 ^C^	795.2 ^A^	0.47	<0.001
ADF (g/kg DM)	459.0 ^B^	457.4 ^B^	443.2 ^B^	478.8 ^A^	0.33	0.0075
ADL (g/kg DM)	81.9 ^B^	80.6 ^B^	73.0 ^C^	98.4 ^A^	0.16	<0.001
GE (MJ/kg DM)	17.4 ^B^	18.0 ^A^	17.8 ^AB^	18.4 ^A^	0.10	<0.001
IVDMd	0.27 ^B^	0.29 ^B^	0.33 ^A^	0.24 ^C^	0.006	<0.001
IVOMd	0.26 ^B^	0.27 ^B^	0.30 ^A^	0.22 ^C^	0.005	<0.001

RF: rangeland fodder, Bruzi: *B. ruziziensis* hay, Pmaxi: *P. maximum* C1 hay, Agaya: *A. gayanus* hay, DM: dry matter, CM= crude matter, OM= Organic matter, CP: crude protein, NDF: neutral detergent fiber, ADF: acid detergent fiber, ADL: acid detergent lignin, GE: gross energy, IVDMd= in vitro dry matter digestibility, IVOMd= in vitro organic matter digestibility.

^A,B,C^ Values within a row with different superscripts differ significantly at *P* < 0.05.

### 2.4 Preprocessing and statistical analyzes

#### 2.4.1 Data preprocessing

Daily individual voluntary intake was calculated as the difference between feed offered and refused. Raw gas exchange data (n = 2320) collected via GF were uploaded to the C-Lock platform. Records were excluded if visit duration was < 3 min, head position was invalid, or sensor proximity was inadequate (< 800 raw), resulting in the removal of 180 data points (≈ 8 %). Spot emissions of eCH₄ and CO₂ (g/d) per visit and animal (n = 2140 per gas) were computed by C-Lock.

Outliers were identified and removed using linear regression, following Coppa et al. (2021), accounting for 0.6 % of the dataset. The final dataset included 2127 records per gas. Visit data were then averaged per animal and day, giving equal weight to day and night periods, to calculate daily individual emissions using Eq. (1):

(Eq. 1) eCH_4_ (g/d) = 0.5 * [(eCH_4_.10:00 *h* + eCH_4_.14:00 *h* + eCH_4_.18:00 h) / 3] + 0.5 * [(eCH_4_.6:30 *h* + eCH_4_.00:00 h) / 2]

with eCH_4_.i: average eCH_4_ emissions (g/d) for visits over the same i period.

The methane conversion rate (Ym, % GEI) was calculated using IPCC (2019) as follows:

(Eq. 2) Ym ( %) = [(eCH_4_ * 55.65 / 1000) /GEI] * 100

where eCH₄ is in g/d and GEI in MJ/d, derived from the GE content of feed offered and refused.

Intake, digestibility, and eCH₄ were calculated per animal per day, and averaged over 7 days. The final dataset per variable per trial was n = 10.

#### 2.4.2 Statistical analysis

All statistical analyses were performed using R software version 4.1.2. (R Core Team, 2021). The normality of variables was initially assessed using the Shapiro–Wilk test (Gonzalez-Estrada & Villasenor-Alva, 2013), while homogeneity of variances was evaluated using Levene’s test (Gozde & Osman, 2024). Intake, digestibility, CO_2_ and eCH_4_ data were analysed using a general linear mixed-effects model (GLM) with the lme4 (Bates et al., 2015) and lmerTest (Kuznetsova et al., 2017) packages. Forage type and feed level (2.3 % vs. 3.2 % BW) were included as fixed effects, and individual animal was treated as a random effect to account for inter-animal variability. Least squares means and their standard errors (SEM) were computed for each factor. When treatment effects were significant (P < 0.05), means were separated using Duncan’s multiple range test (duncan.test, de Mendiburu, 2023). The model used was:

Y_abk_ = μ + T_a_ + Q_b_ + (1 ∣ A_k_) + ϵ_abk_

where Y_abk_ = dependent variable (offered feed quality, intake, digestibility, CO_2_, eCH_4_);

μ = overall mean;

T_a_ = a^th^ effect of forage type (RF, Bruzi, Pmaxi, Agaya);

Q_b_ = b^th^ effect of feed level (2.3 and 3.2 % BW);

A_k_ = random effect of animal (*n*= 10); and

ϵ_abk_ = residual error.

## 3 Results

### 3.1 Chemical component of fodder

All the tested forages were of low nutritional quality due to their advanced harvesting stage. Significant differences were observed among forage types (Table 1). Pmaxi had the highest crude protein (CP) content, while Agaya had the lowest (P < 0.001). Agaya also exhibited the highest NDF content (P < 0.001). The GE content was significantly higher in Bruzi and Agaya (P < 0.001). In vitro digestibility was highest in Pmaxi and lowest in Agaya (P < 0.001).

### 3.2 Forage intake and apparent digestibility

Individual DMI ranged from 12 *g*/kg BW (Bruzi at 2.3 % BW) to 26 *g*/kg BW (Pmaxi at 3.2 % BW). Increasing the forage offer from 2.3 % to 3.2 % BW led to a slight, non-significant increase in intake. Among forages, DMI (g/kg BW) was highest for Pmaxi and lowest for Agaya (P < 0.001; Table 2). Refusal rates were 50.4 %, 47.0 %, 34.2 %, and 53.8 % for RF, Bruzi, Pmaxi, and Agaya, respectively. Refusals averaged 35.8 % at 2.3 % BW and 52.9 % at 3.2 % BW. Overall, forage type significantly influenced intake (P < 0.001). Intake values by forage and feed level are provided in Supplementary Table S1.

**Table 2:** Daily intake of forage hay offered to Sudanese Fulani zebu steers (including bait distributed at the GF).

	Forage type				Offered quantity		SEM	p-value		
	RF	Bruzi	Pmaxi	Agaya	2.3 % BW	3.2 % BW		Forage type	Offered quantity	Forage type x Offered quantity
DMI (g/d)	2419 ^B^	2280 ^B^	3195 ^A^	1913 ^C^	2377	2526	65.6	<0.001	0.348	0.261
DMI (g/kg BW)	16.6 ^B^	16.3 ^B^	20.7 ^A^	14.1 ^C^	16.4	17.4	0.35	<0.001	0.258	0.122
OMI (g/d)	2158 ^B^	2058 ^B^	2894 ^A^	1713 ^C^	2124	2288	60.1	<0.001	0.218	0.583
OMI (g/kg BW)	14.8 ^B^	14.7 ^B^	18.7 ^A^	12.6 ^C^	14.6	15.7	0.32	<0.001	0.134	0.378
CPI (g/d)	68.4 ^C^	84.3 ^B^	121.5 ^A^	35.2 ^D^	73.4	81.3	3.8	<0.001	0.407	0.024
CPI (g/kg BW)	0.47 ^C^	0.60 ^B^	0.78 ^A^	0.26 ^D^	0.50	0.55	0.023	<0.001	0.271	0.002
NDFI (g/d)	1761 ^B^	1557 ^C^	2311 ^A^	1410 ^C^	1711	1808	48.2	<0.001	0.407	0.608
NDFI (g/kg BW)	12.1 ^AB^	11.1 ^B^	15.0 ^A^	10.4 ^B^	11.8	12.4	0.93	<0.001	0.300	0.486
ADFI (g/d)	1100 ^AB^	884 ^B^	1417 ^A^	818 ^B^	1015	1094	31.8	<0.001	0.236	0.940
ADFI (g/kg BW)	7.5 ^AB^	6.3 ^B^	9.2 ^A^	6.0 ^B^	7.0	7.5	0.17	<0.001	0.133	0.688
GEI (MJ/d)	43.3 ^B^	41.0 ^B^	58.7 ^A^	35.0 ^C^	42.7	46.4	1.21	<0.001	0.124	0.649
GEI (MJ/kg BW)	0.29 ^B^	0.29 ^B^	0.37 ^A^	0.25 ^C^	0.29	0.31	0.050	<0.001	0.069	0.378

RF: rangeland fodder, Bruzi: *B. ruziziensis* hay, Pmaxi: *P. maximum* C1 hay, Agaya: *A. gayanus* hay, BW: body weight, 2.3 % BW: animals fed at 2.3 % of BW, 3.2 % BW: animals fed at 3.2 % of BW.

DMI: dry matter intake, OMI: organic matter intake, CPI: crude protein intake, NDFI: neutral detergent fiber intake, ADFI: acid detergent fiber intake, GEI: gross energy intake.

^A,B,C^ Values within a row with different superscripts differ significantly at *P* < 0.05: comparison among forage types.

Varying the quantity offered had no effect on apparent digestibility (Table 3). Apparent digestibility of DM and OM was highest in Pmaxi and lowest in Agaya (P < 0.05). No significant differences were observed among forages for CP digestibility. NDF digestibility was lowest in Bruzi (P = 0.004). Forage type significantly affected digestibility parameters. ADF and GE digestibility were highest in Pmaxi and lowest in Agaya (P < 0.05). Detailed digestibility data by forage and level of offering are presented in Supplementary Table S2.

**Table 3:** Apparent digestibility of forage (including bait distributed at the GF) offered to Sudanese Fulani zebu steers.

	Forage type				Offered quantity		SEM	p-value		
	RF	Bruzi	Pmaxi	Agaya	2.3 % BW	3.2 % BW		Forage type	Offered quantity	Forage type x Offered quantity
DMd (g/kg DM)	464 ^AB^	480 ^AB^	493 ^A^	450 ^B^	468	474	5.7	0.044	0.950	0.644
OMd (g/kg OM)	497 ^AB^	500 ^AB^	527 ^A^	464 ^B^	489	504	6.1	0.005	0.210	0.414
CPd (g/kg CP)	70	122	183	32	104	100	25.8	0.290	0.743	0.433
NDFd (g/kg NDF)	584 ^A^	546 ^B^	589 ^A^	563 ^AB^	567	573	5.2	0.004	0.562	0.747
ADFd (g/kg ADF)	550 ^AB^	520 ^B^	571 ^A^	524 ^B^	531	551	6.8	0.003	0.282	0.025
GEd (MJ/MJ GE)	497 ^B^	482 ^B^	523 ^A^	474 ^B^	485	503	5.8	0.013	0.103	0.640

RF: rangeland fodder, Bruzi: *B. ruziziensis* hay, Pmaxi: *P. maximum* C1 hay, Agaya: *A. gayanus* hay, BW: body weight, 2.3 % BW: animals fed at 2.3 % of BW, 3.2 % BW: animals fed at 3.2 % of BW.

DMd: dry matter digestibility, OMd: organic matter digestibility, CPd: crude protein digestibility, NDFd: neutral detergent fiber digestibility, ADFd: acid detergent fiber digestibility, GEd: gross energy digestibility.

^A,B,C^ Values within a row with different superscripts differ significantly at *P* < 0.05: comparison among forage types.

### 3.3 Enteric methane emissions

Average daily eCH₄ emissions ranged from 59 to 90 *g*/day, with the lowest and highest values observed in Agaya and Pmaxi, respectively (P < 0.001; Table 4). The eCH₄ yield per unit of DM or OM intake did not differ significantly among treatments, although the highest yield was recorded for Agaya. No significant differences were observed in the proportion of GE lost as eCH₄ (Ym). Forage type significantly affected eCH₄ emissions (P < 0.001), while increasing DM offered from 2.3 % to 3.2 % BW had no effect.

**Table 4:** Daily enteric methane emissions by Sudanese Fulani zebu steers fed with different forages.

	Forage type				Offered quantity		SEM	p-value		
	RF	Bruzi	Pmaxi	Agaya	2.3 % BW	3.2 % BW		Forage type	Offered quantity	Forage type x Offered quantity
eCH_4_ (g/d)	72.7 ^B^	68.8 ^B^	90.2 ^A^	59.1 ^C^	71.3	74.0	2.08	<0.001	0.519	0.918
eCH_4_ (g/kg BW)	0.50 ^B^	0.49 ^B^	0.58 ^A^	0.44 ^C^	0.49	0.51	0.013	<0.001	0.480	0.927
eCH_4_ (g/kg DMI)	30.5	30.2	28.5	31.6	30.6	29.8	0.711	0.486	0.565	0.750
eCH_4_ (g/kg dDMI)	66.2 ^AB^	63.2 ^AB^	58.3 ^B^	73.3 ^A^	67.0	63.5	2.08	0.046	0.602	0.817
eCH_4_ (g/kg OMI)	34.2	33.4	31.4	35.3	34.3	32.8	0.80	0.363	0.351	0.915
eCH_4_ (g/kg dOMI)	69.1 ^AB^	67.3 ^AB^	60.0 ^B^	80.3 ^A^	72.6 ^a^	65.7 ^b^	2.36	0.040	0.030	0.680
Ym ( % GEI)	9.5	9.3	8.6	9.6	9.5	9.0	0.22	0.369	0.251	0.878

RF: rangeland fodder, Bruzi: *B. ruziziensis* hay, Pmaxi: *P. maximum* C1 hay, Agaya: *A. gayanus* hay BW: body weight, 2.3 % BW: animals fed at 2.3 % of BW, 3.2 % BW: animals fed at 3.2 % of BW.

eCH_4_: enteric methane, BW: bodyweight, DMI: dry matter intake, dDMI: digestible dry matter intake, OMI: organic matter intake, dOMI: digestible organic matter intake.

^A,B,C^ Values within a row with different superscripts differ significantly at *P* < 0.05: comparison among forage types.

^a,b^ Values within a row with different superscripts differ significantly at *P* < 0.05: comparison between offered quantities.

When extrapolated to an annual basis, eCH₄ emissions per Tropical Livestock Unit (TLU) were estimated at 45.6, 44.7, 52.9, and 40.2 kg/year for RF, Bruzi, Pmaxi, and Agaya, respectively (P < 0.05). Emission values by forage and feeding level are detailed in Supplementary Table S3.

## 4 Discussions

### 4.1 Nutritional quality of forages

Cultivated grasses are now key components of feed diversity in sub-Saharan Africa (SSA). The three grasses tested in this study are among the most widely adopted by farmers in the region and frequently used in scientific research (Klein et al., 2014; Diatta et al., 2019). Assessing their nutritional quality relative to rangeland fodder (RF) is essential. All forages, including RF, were harvested at full maturity, a critical factor in interpreting the results, since this stage is marked by increased lignification (notably cellulose and lignin) and reduced CP content (Gbenou et al., 2024b). Although this uniform maturity supports fair comparison, it underrepresents the species’ peak nutritional potential had they been harvested earlier. The late physiological stage reflects the practical feeding conditions of the dry season, where digestibility and nitrogen content are naturally constrained (Davis et al., 2025; Fernández‐Habas et al., 2025). Using RF under similar conditions enhances the relevance of the findings for extensive Sahelian livestock systems.

The nutritional value of the tested forages varied significantly by type. Pmaxi had the highest CP content, likely due to its lower stalk density. The composition of the RF in this study (CP: 31 *g*/kg DM, NDF: 731 *g*/kg DM, ADF: 476 *g*/kg DM) was comparable to RF values reported for the hot dry season in dry tropical zones (INRA, 2018). However, Pmaxi used in this study, harvested post-flowering, showed lower nutritional quality than Pmaxi hay (CL Orstom variety) harvested at flowering onset (INRA, 2018; Buthelezi et al., 2022). This reflects typical field conditions and explains the reduced CP levels. Pmaxi remained the most nutritious among the cultivated grasses, as also reported by Fernandes et al. (2020). Sokupa et al. (2023) found it to have the highest CP and NDF at 14 weeks post-planting. Soil fertility also plays a role in forage quality; our plots were not fertilized, and fertilization practices in INRA (2018) are undocumented, possibly contributing to the observed differences.

Agaya’s nutritional profile at maturity (CP: 21.6 *g*/kg DM, NDF: 752 *g*/kg DM, ADF: 415 *g*/kg DM) matched previous reports for hay harvested at 120 days (Ribeiro et al., 2014). The typical reduction in CP with increased fiber, leading to lower digestibility, was evident, especially in Agaya (Assoumaya, Sauvant, & Archimède, 2007). Nevertheless, all tested forages exceeded the 7 % CP threshold recommended for ruminant maintenance (NRC, 2001). Higher fiber contents (NDF, ADF) were negatively correlated with in vitro digestibility, aligning with findings by Gbenou et al. (2024b). The lower DM digestibility observed for Agaya compared to rangeland fodder in Kenya (Ndung’u et al., 2020) likely reflects regional differences in forage composition. While *Andropogon* dominates RF in SSA (Ouédraogo et al., 2021), the RF in this study also included *Hyparrhenia* and *Pennisetum*, which may reduce digestibility.

Organic matter (OM) digestibility showed a positive correlation with GE digestibility. For Pmaxi, OM digestibility matched values reported by INRA (2018) for 8-week regrowths of the K187 B Orstom variety, and GE digestibility exceeded those reported by Johnson, Ordoveza, Hardison, and Castillo (1967). Bruzi displayed lower OM digestibility than *B. mutica* (Melesse, Steingass, Schollenberger, & Rodehutscord, 2017). These differences likely stem from genotype, regrowth age, and soil fertility. Overall, the findings highlight the need to explore trade-offs between biomass yield, phenology, and forage quality under late harvesting conditions typical of dry season feeding systems in SSA.

### 4.2 Forage intake

Feed intake remained low across all treatments due to the poor quality of the forages used. Even at 2.3 % BW, refusals were high (35.8 %), consistent with ad libitum conditions (Boval et al., 1996). Increasing the allowance to 3.2 % BW did not significantly increase intake, likely due to digestive constraints associated with mature, high-lignin forages that prolong rumen retention and limit intake capacity (Beckers, 2013; Fanchone et al., 2012). This plateau effect is typical of tropical dry-season forages rich in structural carbohydrates.

The DMI values were consistent with those reported by Assouma et al. (2018) under grazing conditions. Pmaxi showed the highest DMI, attributed to its higher CP content and lower fiber fraction, while Agaya had the lowest intake due to its high fiber content. Bruzi and RF displayed intermediate DMI, reflecting their moderate NDF and CP levels. Only Pmaxi met the 20 *g*/kg BW threshold for tropical cattle intake (Boudet & Rivière, 1968). Leaf texture also favoured Pmaxi, which has thinner, more palatable foliage compared to the stem-dense alternatives.

DMI values for RF, Bruzi, and Agaya (15 - 18 *g*/kg BW) aligned with those reported by Gbenou et al. (2024b), highlighting their low nutritive value. Beyond intake, Pmaxi’s potential for intensive grazing (Tesk et al., 2018) enhances its agronomic appeal. Apparent digestibility was highest for Pmaxi and lowest for Agaya, consistent with their CP and fiber profiles. Protein-rich forages promote greater digestibility through enhanced fermentability and volatile fatty acid production (Tovignon et al., 2021). Digestibility of DM and OM correlated positively with in vitro measures, as also noted by Geisert et al. (2007). Forage type significantly affected both intake and digestibility, though within-forage comparisons showed significant DMI differences only for Pmaxi across supply levels.

### 4.3 Enteric methane emissions

While the four tested forages have been widely studied in ruminant feeding in SSA (Dias et al., 2022; Gbenou et al., 2020; Ogunbosoye & Odedire, 2022), their environmental impact, particularly eCH₄ emissions, has received limited attention. This study addressed that gap, highlighting forage-specific effects on eCH₄ emissions.

Forage quality and type significantly influence eCH₄ production by affecting ruminal digestibility and fermentation dynamics. Pmaxi led to the highest absolute eCH₄ production, consistent with its superior intake and digestibility. This aligns with findings by Alvarado-Bolovich et al. (2021), who reported higher eCH₄ emissions with more digestible and highly consumed forages. Higher intake of Pmaxi enhances the rate of passage, accelerating digesta flow through the rumen. In contrast, Agaya was less ingested and less digestible due to its lower nutritional quality. Despite Pmaxi showing the highest total methane output, the variation in ruminal passage rates across forage types was insufficient to elicit a statistically significant difference in eCH_4_ yield per unit of dry matter (g /kg DMI). Nevertheless, the eCH₄ yield from Pmaxi was lower when expressed per unit of digestible matter, indicating greater eco-efficiency. However, confirmation requires further studies at different growth stages. Increasing DM allowance from 2.3 % to 3.2 % BW had no effect on eCH₄ output, likely due to limited differences in actual intake, consistent with Gbenou et al. (2024a).

Annual eCH₄ emissions ranged from 40.15 to 52.92 kg/TLU, with only Pmaxi approximating the IPCC (2019) Tier 1 default of 52 kg/TLU/year. These discrepancies are partly methodological: Tier 1 estimates aggregate adult and growing cattle, whereas our results reflect growing steers only. Our findings also align with Gurmu et al. (2024), who reported 46 kg/TLU/year for steers fed low-quality forages. The yields observed (28–32 *g*/kg DMI) fall within ranges reported in SSA: e.g. 29.1 *g*/kg DMI in Jersey cows on *Pennisetum clandestinum* pasture (van Wyngaard et al., 2018), and 30.6 *g*/kg DMI in Holstein × Boran heifers on wheat stalks (Ali et al., 2019). These values contrast with lower yields reported by Gbenou et al. (2024a, 2024b) for steers on natural forages or supplemented diets. Such variability underscores the influence of forage type, maturity stage, and diet composition on eCH₄ emissions.

The average yield of 30.2 *g*/kg DMI (72.7 *g*/day) exceeded the IPCC (2019) Tier 2 default (23.3 *g*/kg DMI) for growing animals on low-digestibility, forage-based diets. Higher emissions observed elsewhere, e.g., 77.8–178 *g*/day in steers across Zimbabwe, Ethiopia, and Benin (Gurmu et al., 2024; Gwatibaya et al., 2023; Kouazounde et al., 2015; Ndung’u et al., 2022), further illustrate variation driven by breed, diet, and measurement method. While none of the cultivated forages showed clear environmental superiority, Pmaxi emerges as a potential candidate due to its lower emissions per unit of digestible matter intake. However, further research is needed across vegetative stages over longer durations, taking into account the physiological stages of cattle, to validate this trend and develop region-specific emission factors.

## 5 Conclusion

Forage diversity in Sahelian livestock production systems supports feed supply, animal performance, and farmer profitability. It is well-established that harvesting at maturity increases fiber content and reduces protein levels. Among the four low-quality forages tested, *Andropogon gayanus* was the most fibrous and least consumed, while *Panicum maximum* C1 had higher protein content and was the most ingested. Increasing feed allowance beyond ad libitum had no effect on intake, digestibility, or enteric methane emissions. All forages produced similar methane yields. These findings suggest that enhancing feed nutritional quality, through earlier harvesting or protein supplementation, is crucial for improving intake and digestibility, while also mitigating emissions during the dry season, without requiring significant changes to existing practices. However, optimization efforts must also account for forage seasonality and availability constraints typical of Sahelian livestock production systems.

## Ethics approval

This study was approved by the CIRDES Ethics Committee for Animal Experiments under application number 006/Mars/2021/CE CIRDES, dated 15 April 2021.

## Data and model availability statement

The datasets generated and analyzed during the current study are not publicly available but are available from the corresponding author on reasonable request.

## Declaration of generative AI and AI-assisted technologies in the writing process

During the preparation of this work, the author(s) used AI-assisted technologies for English editing purposes, including checking for grammatical errors and correcting misplaced punctuation.

## Financial support statement

This study was made possible through the support of the ‘‘Carbon Sequestration and greenhouse gas emissions in (agro) Sylvopastoral Ecosystems in the sahelian CILSS States” (CaSSECS) regional project funded by the European Union (European DeSIRA programme, under grant agreement No FOOD/2019/410–169).

## Ethical statement

All experimental procedures involving animals were conducted in accordance with institutional guidelines and regulations for the ethical treatment of animals. The study titled "Enteric Methane Emissions in Zebu Cattle Fed Low-Quality Forages in Sahel" was reviewed and approved by the Ethics Committee for Animal Experiments of the Centre International de Recherche-Développement sur l’Élevage en zone Subhumide (CIRDES), under application number 006/Mars/2021/CE CIRDES, dated 15 April 2021.

Gérard Xavier GBENOU, Luc Hippolyte DOSSA, Denis BASTIANELLI, Ollo SIB, Laurent BONNAL, Cécile MARTIN, Mohamed Habibou ASSOUMA

## CRediT authorship contribution statement

**Gérard Xavier Gbenou:** Writing – review & editing, Writing – original draft, Formal analysis, Data curation, Conceptualization. **Luc Hippolyte Dossa:** Writing – review & editing, Validation, Supervision, Conceptualization. **Denis Bastianelli:** Writing – review & editing, Writing – original draft, Formal analysis, Conceptualization. **Ollo Sib:** Writing – review & editing, Investigation, Conceptualization. **Laurent Bonnal:** Writing – review & editing, Formal analysis, Data curation. **Cécile Martin:** Writing – review & editing, Supervision, Formal analysis, Conceptualization. **Mohamed Habibou Assouma:** Writing – review & editing, Writing – original draft, Validation, Supervision, Project administration, Methodology, Funding acquisition, Formal analysis, Data curation, Conceptualization.

## Declaration of competing interest

The authors whose names are listed immediately below certify that they have no conflict, NO affiliations with or involvement in any organization or entity with any financial interest (such as honoraria; educational grants; participation in speakers’ bureaus; membership, employment, consultancies, stock ownership, or other equity interest; and expert testimony or patent-licensing arrangements), or non-financial interest (such as personal or professional relationships, affiliations, knowledge or beliefs) in the subject matter or materials discussed in this manuscript. The manuscript represents valid work; neither this manuscript nor one with substantially similar content under our authorship has been published or is being considered for publication elsewhere (except as described in the manuscript submission); and copies of any closely related manuscripts are enclosed in the manuscript submission.

## Appendix Supplementary materials

Image, application 1

## References

[bib0001] Ali A.I.M., Wassie S.E., Korir D., Merbold L., Goopy J.P., Butterbach-Bahl K., Dickhoefer U., Schlecht V. (2019). Supplementing tropical cattle for improved nutrient utilization and reduced enteric methane emissions. Animals.

[bib0002] Alvarado-Bolovich V., Medrano J., Haro J., Castro-Montoya J., Dickhoefer U., Gomez C. (2021). Enteric methane emissions from lactating dairy cows grazing cultivated and native pastures in the high Andes of Peru Víctor. Livestock Science.

[bib45] Amole T., Augustine A., Balehegn M., Adesogoan A.T. (2022). Livestock feed resources in the West African Sahel. Agronomy Journal.

[bib0003] Assouma M.H., Lecomte P., Hiernaux P., Ickowicz A., Corniaux C., Decruyenaere V., Diarra A.R., Vayssieres J. (2018). How to better account for livestock diversity and fodder seasonality in assessing the fodder intake of livestock grazing semi-arid sub-Saharan Africa rangelands. Livestock Science.

[bib50] Assoumaya C., Sauvant D., Archimède H. (2007). Comparative study of intake and digestion of tropical and temperate forages. INRAE Productions Animales.

[bib0004] Aufrère J., Baumont R., Delaby L., Peccatte J.R., Andrieu J., Andrieu J.-P., Dulphy J.P. (2007). Laboratory prediction of forage digestibility by the pepsin-cellulase method. The renewed equations. INRAE Productions Animales.

[bib0005] Bates D., Mächler M., Bolker B., Walker S. (2015). Fitting linear mixed-effects models using lme4. Journal of Statistical Software.

[bib0006] Beckers Y. (2013). A balanced diet for bovines: Strategies to improve the efficiency of nitrogen use. Biotechnol. Agronomy, Society and Environment.

[bib0007] Boudet G., Rivière R. (1968). Practical use of fodder analysis to assess the value of tropical pastures. REMVT.

[bib0008] Boval M., Peyraud J.l., Xande A., Aumont G., Coppry O., Saminadin G. (1996). Evaluation of faecal indicators to predict digestibility and voluntary intake of Dichanthium sp by cattle. Annales de Zootechnie.

[bib0009] Buthelezi L.S., Mupangwa J., Washaya S. (2022). Fodder pro-duction and chemical composition of pigeon pea [Cajanus cajan [Millspaugh] varieties grown in the subtropical region of South Africa. Tropical and Subtropical Agroecosystems.

[bib0010] Coppa M., Jurquet J., Eugène M., Dechaux T., Rochette Y., Lamy J., Ferlay A., Martin C. (2021). Repeatability and ranking of long-term enteric methane emissions measurement on dairy cows across diets and time using GreenFeed system in farm-conditions. Methods.

[bib0011] Davis N.G., Wyffels S.A., DelCurto T. (2025). Influence of temperature and precipitation on the forage quality of Bluebunch Wheatgrass and Idaho Fescue during the dormant season. Grasses.

[bib47] de Mendiburu F. (2023). R package version 1.3-3.

[bib0013] Dias M.B.C., Costa K.A.P., Severiano E.C., Bilego U.O., Vilela L., de Souza W.F., de Oliveira I.P., da Silva A.C.G. (2022). Cattle performance with Brachiaria and Panicum maximum forages in an integrated crop-livestock system. African Journal Of Range & Forage Science.

[bib0014] Diatta A., Thiam M.B., Traore E.H. (2019). Recherches sur les cultures fourragères au Sénégal: Quelques résultats. Journée Nationale de l’Elevage.

[bib0015] Diawara M.O., Hiernaux P., Mougin E., Gangneron F., Soumaguel N. (2017). Visibility of pastoralism in the Sahel: Study of demographic parameters of livestock herds in Hombori (Mali). Cahiers Agricultures.

[bib0016] Fanchone A., Nozière P., Portelli J., Duriot B., Largeau V., Doreau M. (2012). Effects of nitrogen underfeeding and energy source on nitrogen ruminal metabolism, digestion, and nitrogen partitioning in dairy cows. Journal Of Animal Science.

[bib0017] Fernandes L.S., Difante G.S., Costa M.G., Neto J.V.E., de Araújo I.M.M., Dantas J.L.S., Gurgel A.L.C. (2020). Pasture structure and sheep performance supplemented on different tropical grasses in the dry season. Revista Mexicana de Ciencias Pecuarias.

[bib0018] Fernández-Habas J., Real D., Vanwalleghem T., Leal-Murillo J.R., Fernández-Rebollo P. (2025). Yield and forage quality of the new forage perennial legume Bituminaria bituminosa var. albomarginata cv. Lanza in response to rainfall reduction and competition. Journal of Agronomy and Crop Science.

[bib0019] Gbenou G.X., Assouma M.H., Bastianelli D., Kiendrebeogo T., Bonnal L., Zampaligre N., Bois B., Sanogo S., Sib O., Martin C., Dossa L.H. (2024). Enteric methane emissions from zebu cattle in Sub-Saharan Africa are influenced by seasonal variations in rangeland fodder quality and intake. Animal : An International Journal Of Animal Bioscience.

[bib0020] Gbenou G.X., Assouma M.H., Bastianelli D., Kiendrebeogo T., Bonnal L., Zampaligre N., Bois B., Sanogo S., Sib O., Martin C., Dossa L.H. (2024). Supplementing zebu cattle with crop co-products helps to reduce enteric emissions in West Africa. Archives of Animal Nutrition.

[bib0021] Gbenou G.X., Soule H.A., Akpo Y., Djenontin A.J.P., Kperou Gado B.O., Babatounde S., Houinato M., Sidi H., Mensah G.A. (2020). Dairy and economic performances of mixed cows (gir x borgou) complemented with sorgho brewer’s on Panicum maximum c1 grazing in Northen Benin. Agron. Afr..

[bib0022] Geisert B.G., Klopfenstein T.J., Adams D.C., MacDonald J.C. (2007). https://digitalcommons.unl.edu/animalscinbcr/95.

[bib0023] Gonzalez-Estrada E., Villasenor-Alva J.A. (2013). https://cran.r-project.org/web/packages/mvShapiroTest/mvShapiroTest.pdf.

[bib0025] Gozde C., Osman D. (2024). https://cran.r-project.org/web/packages/vartest/vartest.pdf.

[bib0026] Gurmu E.B., Ndung'u P.W., Wilkes A., Getahun D., Graham M.W., Leitner S.M., Marquardt S., Mulatb D.G., Merbolde L., Workuf T., Kagai J.G., Arndt C. (2024). Comparison of Tier 1 and 2 methodologies for estimating intake and enteric methane emission factors from smallholder cattle systems in Africa: A case study from Ethiopia. Animal-Open Space.

[bib0027] Gwatibaya S., Murungweni C., Mpofu I., Jingura R., Tigere A.T., Tererai B. (2023). Enteric methane emission estimates for the Zimbabwean Sanga cattle breeds of Tuli and Mashona. Tropical Animal Health And Production.

[bib0028] INRA, 2018. INRA feeding system for ruminants: Specific feeding of ruminant in hot regions. Editions Quæ, Versailles, France, 640p 10.3920/978-90-8686-292-4.

[bib0029] Calvo Buendia E., Tanabe K., Kranjc A., Baasansuren J., Fukuda M., Ngarize S., Osako A., Pyrozhenko Y., Shermanau P., Federici S., IPCC (2019). 2019 Refinement to the 2006 ipcc guidelines for national greenhouse gas inventories.

[bib53] Johnson W.L., Ordoveza A.L., Hardison W.A., Castillo L.S. (1967). The nutritive value of Panicum maximum (Guinea grass) II. Digestibility by cattle and water buffaloes, related to season and herbage growth stage. The Journal of Agricultural Science.

[bib0030] Kering M.K., Rahemi A., Temu V.W. (2024). Effect of harvest management on biomass yield, forage quality, and nutrient removal by bioenergy grasses in mid-central Virginia. Agronomy.

[bib0031] Kiema A., Tontibomma G.B., Zampaligré N. (2014). Transhumance et gestion des ressources naturelles au Sahel: Contraintes et perspectives face aux mutations des systèmes de productions pastorales. VertigO - La Revue Électronique En Sciences De L'environnement.

[bib0032] Klein, H.D., Rippstein, G., Huguenin, J., Toutain, B., Guerin, H., Louppe, D., 2014. Les cultures fourragères, agricultures tropicales en poche, Éditions Quæ, ISBN (Quæ): 978-2-7592-2168-4, 264p.

[bib0033] Kouazounde J.B., Gbenou J.D., Babatounde S., Srivastava N., Eggleston S.H., Antwi C., Baah J., McAllister T.A. (2015). Development of methane emission factors for enteric fermentation in cattle from Benin using IPCC Tier 2 methodology. Animal : An International Journal Of Animal Bioscience.

[bib0034] Koutou M., Sangaré M., Havard M., Vall E., Sanogo L., Thombiano T., Vodouhe D.S. (2016). Diversity of breeding practices in western cotton zone of Burkina Faso. Agronomie Africaine.

[bib0035] Kuznetsova A., Brockhoff P.B., Christensen R.H.B. (2017). lmerTest package: Tests in linear mixed effects models. Journal of Statistical Software.

[bib54] Melesse A., Steingass H., Schollenberger M., Rodehutscord M. (2017). Screening of common tropical grass and legume forages in Ethiopia for their nutrient composition and methane production profile in vitro. Tropical Grasslands-Forrajes Tropicales.

[bib0036] Meo-Filho P., Hood J., Lee M.R.F., Fleming H., Meethal M.E., Misselbrook T. (2023). Performance and enteric methane emissions from housed beef cattle fed silage produced on pastures with different forage profiles. Animal : An International Journal Of Animal Bioscience.

[bib52] Ndung’u P.W., Bebe B.O., Ondiek J.O., Butterbach-Bahl K., Merbold L., Goopy J.P. (2020). Corrigendum to: Improved region-specific emission factors for enteric methane emissions from cattle in smallholder mixed crop: livestock systems of Nandi County, Kenya. Animal Production Science.

[bib56] Ndung’u P.W., du Toit, Takahashi T., Robertson-Dean M., Butterbach Bahl K., Goopy J.P. (2022). A simplified approach for producing Tier 2 enteric-methane emission factors based on East African smallholder farm data. Animal Production Science.

[bib51] NRC (2001). Nutrient Requirements for Dairy Cattle.

[bib0037] Obulbiga M.F., Bougouma V., Sanon H.O. (2015). Amélioration de l’offre fourragère par l’association culturale céréale légumineuse à double usage en zone nord soudanienne du Burkina Faso. International Journal Of Biological And Chemical Sciences.

[bib0038] Ogunbosoye D.O., Odedire J.A. (2022). Evaluation of silage from maize stover, maize husk and Andropogon gayanus in equal level with Tephrosia bracteolata as feed for West African Dwarf sheep. Tropical Animal Health And Production.

[bib0039] Ouédraogo K., Zaré A., Korbéogo G., Ouédraogo O., Linstädter A. (2021). Resilience strategies of West African pastoralists in response to scarce forage resources. Pastoralism: Research, Policy and Practice.

[bib46] R Core Team (2021). R: A language and environment for statistical computing.

[bib49] Ribeiro, Teixeira A.M., Velasco F.O., Júnior W.F., Pereira L.G.R., McAllister T.A. (2014). Production, nutritional quality and in vitro methane production from Andropogon gayanus grass harvested at different maturities and preserved as hay or silage. Asian-Australasian Journal of Animal Sciences.

[bib0041] Sokupa M.I., Mupangwa J.F., Washaya S., Tikwayo S.E., Mopipi K. (2023). The nutritive value of Panicum maximum and, Brachiaria brizantha grass species, Acta Agriculturae Scandinavica, Section A. Animal Science.

[bib55] Tesk C.R.M., Pedreira B.C., Pereira D.H., Pina D.D.S., Ramos T.A., Mombach M.A. (2018). Impact of grazing management on forage qualitative characteristics: a review. Scientific Electronic Archives.

[bib0043] Tovignon G.C.Z., Toure A.I., Kimse M., Thiam M., Engonga L.C., Boukila B. (2021). Effects of the incorporation of Pueraria phaseoloides on growth performance of rabbit (Oryctolagus cuniculus). Afrique Science.

[bib0044] van Wyngaard J.D.V., Meeske R., Erasmus L.J. (2018). Effect of concentrate level on enteric methane emissions, production performance, and rumen fermentation of Jersey cows grazing kikuyu-dominant pasture during summer. Journal Of Dairy Science.

