# Supplementary material for: Nutrients utilization and enteric methane emission in zebu cattle fed low quality forages
Source: Vet Anim Sci. 2025 Sep 18;30:100511. doi: 10.1016/j.vas.2025.100511 (PMC12494851; doi:10.1016/j.vas.2025.100511)
Supplement: Supplementary file 1 [file mmc1.docx]

**NUTRIENTS UTILIZATION AND ENTERIC METHANE EMISSION IN ZEBU CATTLE FED LOW QUALITY FORAGES**

Gérard Xavier GBENOU^1.2^, Luc Hippolyte DOSSA^1^, Denis BASTIANELLI^2^, Ollo SIB^2.3.4^, Laurent BONNAL^2^, Cécile MARTIN^5^, Mohamed Habibou ASSOUMA^2.3.4^

^1^Laboratoire des Sciences Animales, Faculté des Sciences Agronomiques, Université d’Abomey-Calavi, 526 Cotonou, Benin

^2^SELMET, University of Montpellier, CIRAD, INRAE, Institut Agro, Montpellier, France

^3^CIRAD, UMR SELMET, dP ASAP, Bobo Dioulasso, Burkina Faso

^4^Centre International de Recherche Développement sur l’Elevage en zone Subhumide, 454 Bobo-Dioulasso, Burkina Faso

5Université Clermont Auvergne, INRAE, VetAgro Sup, UMR 1213 Herbivores, 63122 Saint-Genès-Champanelle, France

Corresponding author : [habibou.assouma@cirad.fr](mailto:habibou.assouma@cirad.fr)

# Supplementary File

Additional data for this study can be found in the following tables.

**Table S1**

Effect of forage offer levels on daily dry matter intake in Sudanese Fulani zebu steers (including bait distributed at the GF)

|  | **RF** | | | | **Bruzi** | | | | **Pmaxi** | | | | **Agaya** | | | |
| --- | --- | --- | --- | --- | --- | --- | --- | --- | --- | --- | --- | --- | --- | --- | --- | --- |
|  | 2.3% BW | 3.2% BW | SEM | P-value | 2.3% BW | 3.2% BW | SEM | P-value | 2.3% BW | 3.2% BW | SEM | P-value | 2.3% BW | 3.2% BW | SEM | P-value |
| DMI (kg) | 2.4 | 2.4 | 0.07 | 0.911 | 2.1 | 2.4 | 0.08 | 0.094 | 3.0 B | 3.3 A | 0.09 | 0.028 | 1.9 | 1.9 | 0.05 | 0.994 |
| DMI (g/kg LW) | 16.8 | 16.4 | 0.45 | 0.648 | 15.3 | 17.2 | 0.54 | 0.076 | 19.6 B | 21.7 A | 0.54 | 0.047 | 13.9 | 14.2 | 0.21 | 0.500 |
| OMI (kg/d) | 2.1 | 2.2 | 0.07 | 0.911 | 1.9 | 2.2 | 0.07 | 0.126 | 2.8 B | 3.0 A | 0.09 | 0.028 | 1.7 | 1.8 | 0.05 | 0.409 |
| OMI (g/kg LW) | 14.8 | 14.8 | 0.39 | 0.993 | 13.9 | 15.5 | 0.49 | 0.100 | 17.8 | 19.6 | 0.48 | 0.062 | 12.1 | 13.0 | 0.23 | 0.038 |
| CPI (kg) | 0.06 | 0.06 | 0.002 | 0.100 | 0.08 | 0.08 | 0.002 | 0.795 | 0.10 A | 0.13 A | 0.004 | 0.016 | 0.02 B | 0.04 A | 0.001 | <0.001 |
| CPI (g/kg LW) | 0.47 | 0.46 | 0.014 | 0.731 | 0.60 | 0.60 | 0.014 | 0.972 | 0.72 B | 0.84 A | 0.023 | 0.005 | 0.21 B | 0.30 A | 0.013 | <0.001 |
| NDFI (kg) | 1.7 | 1.8 | 0.06 | 0.578 | 1.5 | 1.6 | 0.06 | 0.166 | 2.2 | 2.4 | 0.06 | 0.075 | 1.4 | 1.4 | 0.00 | 0.931 |
| NDFI (g/kg LW) | 12.0 | 12.1 | 0.33 | 0.930 | 10.5 | 11.7 | 0.38 | 0.142 | 14.4 | 15.5 | 0.37 | 0.147 | 10.3 | 10.4 | 0.19 | 0.703 |
| ADFI (kg) | 1.1 | 1.1 | 0.03 | 0.606 | 0.8 | 0.9 | 0.03 | 0.216 | 1.4 | 1.5 | 0.04 | 0.075 | 0.8 | 0.9 | 0.00 | 0.160 |
| ADFI (g/kg LW) | 7.5 | 7.6 | 0.20 | 0.792 | 6.0 | 6.6 | 0.22 | 0.199 | 8.9 | 9.5 | 0.23 | 0.205 | 5.6 B | 6.4 A | 0.15 | 0.009 |
| GEI (MJ) | 42.5 | 44.2 | 1.45 | 0.576 | 38.4 | 43.6 | 1.47 | 0.077 | 55.8 B | 61.7 A | 1.76 | 0.028 | 33.9 | 36.1 | 1.07 | 0.341 |
| GEI (MJ/kg LW) | 0.29 | 0.30 | 0.097 | 0.765 | 0.27 B | 0.31 A | 0.026 | 0.041 | 0.36 | 0.40 | 0.054 | 0.062 | 0.24 | 0.27 | 0.127 | 0.021 |

RF: rangeland fodder, Bruzi: *B. ruziziensis* hay, Pmaxi: *P. maximum* C1 hay, Agaya: *A. gayanus* hay, LW: live weight, DMI: dry matter intake, OMI: organic matter intake, CPI: crude protein intake, NDFI: neutral detergent fiber intake, ADFI: acid detergent fiber intake, GEI: gross energy intake

^A,B^ Values within a row with different superscripts differ significantly at P < 0.05: comparison between forage types

**Table S2**

Effect of forage offer levels on diet apparent digestibility in Sudanese Fulani zebu steers (including bait distributed at the GF)

|  | **RF** | | | | **Bruzi** | | | | **Pmaxi** | | | | **Agaya** | | | |
| --- | --- | --- | --- | --- | --- | --- | --- | --- | --- | --- | --- | --- | --- | --- | --- | --- |
|  | 2.3% BW | 3.2% BW | SEM | P-value | 2.3% BW | 3.2% BW | SEM | P-value | 2.3% BW | 3.2% BW | SEM | P-value | 2.3% BW | 3.2% BW | SEM | P-value |
| DMd | 0.47 | 0.46 | 0.007 | 0.725 | 0.47 | 0.48 | 0.008 | 0.403 | 0.49 A | 0.50 A | 0.010 | 0.655 | 0.44 | 0.46 | 0.015 | 0.454 |
| OMd | 0.49 | 0.50 | 0.007 | 0.656 | 0.49 | 0.51 | 0.009 | 0.319 | 0.53 A | 0.52 A | 0.009 | 0.751 | 0.44 | 0.48 | 0.016 | 0.161 |
| CPd | 0.13 | 0.00 | 0.053 | 0.192 | 0.12 | 0.11 | 0.041 | 0.899 | 0.13 B | 0.22 A | 0.045 | 0.033 | 0.05 | 0.01 | 0.061 | 0.882 |
| NDFd | 0.57 | 0.59 | 0.006 | 0.218 | 0.54 | 0.55 | 0.009 | 0.517 | 0.59 | 0.58 | 0.008 | 0.486 | 0.55 | 0.57 | 0.013 | 0.655 |
| ADFd | 0.54 | 0.56 | 0.007 | 0.254 | 0.52 | 0.51 | 0.011 | 0.790 | 0.58 | 0.56 | 0.009 | 0.408 | 0.48 B | 0.56 A | 0.020 | 0.049 |
| GEd | 0.49 | 0.51 | 0.008 | 0.188 | 0.47 | 0.49 | 0.009 | 0.321 | 0.52 | 0.52 | 0.009 | 0.827 | 0.45 | 0.49 | 0.014 | 0.257 |

RF: rangeland fodder, Bruzi: *B. ruziziensis* hay, Pmaxi: *P. maximum* C1 hay, Agaya: *A. gayanus* hay, DMd: dry matter digestibility, OMd: organic matter digestibility, CPd: crude protein digestibility, NDFd: neutral detergent fiber digestibility, ADFd: acid detergent fiber digestibility, GEd: gross energy digestibility

^A,B^ Values within a row with different superscripts differ significantly at P < 0.05: comparison between forage types

**Table S3**

Effect of forage offer levels on daily enteric methane emissions in Sudanese Fulani zebu steers

|  | **RF** | | | | **Bruzi** | | | | **Pmaxi** | | | | **Agaya** | | | |
| --- | --- | --- | --- | --- | --- | --- | --- | --- | --- | --- | --- | --- | --- | --- | --- | --- |
|  | 2.3% BW | 3.2% BW | SEM | P-value | 2.3% BW | 3.2% BW | SEM | P-value | 2.3% BW | 3.2% BW | SEM | P-value | 2.3% BW | 3.2% BW | SEM | P-value |
| eCH_4_ (g) | 72.2 | 73.2 | 3.48 | 0.882 | 68.5 | 69.0 | 3.28 | 0.932 | 86.8 | 93.6 | 3.94 | 0.406 | 57.9 | 60.3 | 2.61 | 0.970 |
| eCH_4_ (g/kg BW) | 0.50 | 0.50 | 0.024 | 0.967 | 0.49 | 0.49 | 0.020 | 0.890 | 0.56 | 0.60 | 0.02 | 0.450 | 0.42 | 0.45 | 0.021 | 0.739 |
| eCH_4_ (g/kg DMI) | 30.3 | 30.6 | 1.63 | 0.931 | 31.8 | 28.5 | 1.00 | 0.096 | 29.0 | 28.0 | 1.19 | 0.681 | 31.2 | 32.0 | 1.75 | 0.832 |
| eCH_4_ (g/kg dDMI) | 66.1 | 66.4 | 3.85 | 0.972 | 67.7 A | 58.7 B | 2.46 | 0.043 | 58.2 | 58.3 | 2.91 | 0.982 | 76.1 A | 70.5 B | 6.00 | 0.043 |
| eCH_4_ (g/kg OMI) | 34.4 | 33.9 | 1.81 | 0.880 | 35.2 | 31.7 | 1.13 | 0.125 | 31.8 | 30.9 | 1.29 | 0.735 | 35.8 | 34.8 | 1.99 | 0.803 |
| eCH_4_ (g/kg dOMI) | 67.5 | 70.8 | 4.02 | 0.697 | 72.1 A | 62.5 B | 2.63 | 0.047 | 60.1 | 59.9 | 2.82 | 0.969 | 87.5 A | 73.0 B | 7.09 | 0.037 |
| Ym (% GEI) | 9.6 | 9.3 | 0.50 | 0.767 | 9.9 | 8.8 | 0.32 | 0.075 | 8.7 | 8.5 | 0.35 | 0.737 | 9.8 | 9.4 | 0.53 | 0.764 |

RF: rangeland fodder, Bruzi: *B. ruziziensis* hay, Pmaxi: *P. maximum* C1 hay, Agaya: *A. gayanus* hay, BW: body weight, eCH_4_: enteric methane, DMI: dry matter intake, dDMI: digestible dry matter intake, OMI: organic matter intake, dOMI: digestible organic matter intake

^A,B^ Values within a row with different superscripts differ significantly at P < 0.05: comparison between forage types
